# Potential to reduce greenhouse gas emissions through different dairy cattle systems in subtropical regions

**DOI:** 10.1371/journal.pone.0234687

**Published:** 2020-06-18

**Authors:** Henrique M. N. Ribeiro-Filho, Maurício Civiero, Ermias Kebreab

**Affiliations:** 1 Department of Animal Science, University of California, Davis, California, United States of America; 2 Programa de Pós-graduação em Ciência Animal, Universidade do Estado de Santa Catarina, Lages, Santa Catarina, Brazil; USDA Agricultural Research Service, UNITED STATES

## Abstract

Carbon (C) footprint of dairy production, expressed in kg C dioxide (CO_2_) equivalents (CO_2_e) (kg energy-corrected milk (ECM))^-1^, encompasses emissions from feed production, diet management and total product output. The proportion of pasture on diets may affect all these factors, mainly in subtropical climate zones, where cows may access tropical and temperate pastures during warm and cold seasons, respectively. The aim of the study was to assess the C footprint of a dairy system with annual tropical and temperate pastures in a subtropical region. The system boundary included all processes up to the animal farm gate. Feed requirement during the entire life of each cow was based on data recorded from Holstein × Jersey cow herds producing an average of 7,000 kg ECM lactation^-1^. The milk production response as consequence of feed strategies (scenarios) was based on results from two experiments (warm and cold seasons) using lactating cows from the same herd. Three scenarios were evaluated: total mixed ration (TMR) *ad libitum* intake, 75, and 50% of *ad libitum* TMR intake with access to grazing either a tropical or temperate pasture during lactation periods. Considering IPCC and international literature values to estimate emissions from urine/dung, feed production and electricity, the C footprint was similar between scenarios, averaging 1.06 kg CO_2_e (kg ECM)^-1^. Considering factors from studies conducted in subtropical conditions and actual inputs for on-farm feed production, the C footprint decreased 0.04 kg CO_2_e (kg ECM)^-1^ in scenarios including pastures compared to *ad libitum* TMR. Regardless of factors considered, emissions from feed production decreased as the proportion of pasture went up. In conclusion, decreasing TMR intake and including pastures in dairy cow diets in subtropical conditions have the potential to maintain or reduce the C footprint to a small extent.

## Introduction

Greenhouse gas (GHG) emissions from livestock activities represent 10–12% of global emissions [1], ranging from 5.5–7.5 Gt CO_2_ equivalents (CO_2_e) yr^-1^, with almost 30% coming from dairy cattle production systems [2]. However, the livestock sector supply between 13 and 17% of calories and between 28 and 33% of human edible protein consumption globally [3]. Additionally, livestock produce more human-edible protein per unit area than crops when land is unsuitable for food crop production [4].

Considering the key role of livestock systems in global food security, several technical and management interventions have been investigated to mitigate methane (CH_4_) emissions from enteric fermentation [5], animal management [6] and manure management [7]. CH_4_ emissions from enteric fermentation represents around 34% of total emissions from livestock sector, which is the largest source [2]. Increasing proportions of concentrate and digestibility of forages in the diet have been proposed as mitigation strategies [1,5]. In contrast, some life cycle assessment (LCA) studies of dairy systems in temperate regions [8–11] have identified that increasing concentrate proportion may increase carbon (C) footprint due to greater resource use and pollutants from the production of feed compared to forage. Thus, increasing pasture proportion on dairy cattle systems may be an alternative management to mitigate the C footprint.

In subtropical climate zones, cows may graze tropical pastures rather than temperate pastures during the warm season [12]. Some important dairy production areas, such as southern Brazil, central to northern Argentina, Uruguay, South Africa, New Zealand and Australia, are located in these climate zones, having more than 900 million ha in native, permanent or temporary pastures, producing almost 20% of global milk production [13]. However, due to a considerable inter-annual variation in pasture growth rates [14,15], the interest in mixed systems, using total mixed ration (TMR) + pasture has been increasing [16]. Nevertheless, to our best knowledge, studies conducted to evaluate milk production response in dairy cow diets receiving TMR and pastures have only been conducted in temperate pastures and not in tropical pastures (e.g. [17–19]).

It has been shown that dairy cows receiving TMR-based diets may not decrease milk production when supplemented with temperate pastures in a vegetative growth stage [18]. On the other hand, tropical pastures have lower organic matter digestibility and cows experience reduced dry matter (DM) intake and milk yield compared to temperate pastures [20,21]. A lower milk yield increases the C footprint intensity [22], offsetting an expected advantage through lower GHG emissions from crop and reduced DM intake.

The aim of this work was to quantify the C footprint and land use of dairy systems using cows with a medium milk production potential in a subtropical region. The effect of replacing total mixed ration (TMR) with pastures during lactation periods was evaluated.

## Materials and methods

An LCA was developed according to the ISO standards [23,24] and Food and Agriculture Organization of the United Nations (FAO) Livestock Environmental Assessment Protocol guidelines [25]. All procedures were approved by the ‘Comissão de Ética no Uso de Animais’ (CEUA/UDESC) on September 15, 2016—Approval number 4373090816 - https://www.udesc.br/cav/ceua.

### System boundary

**Fig 1:**
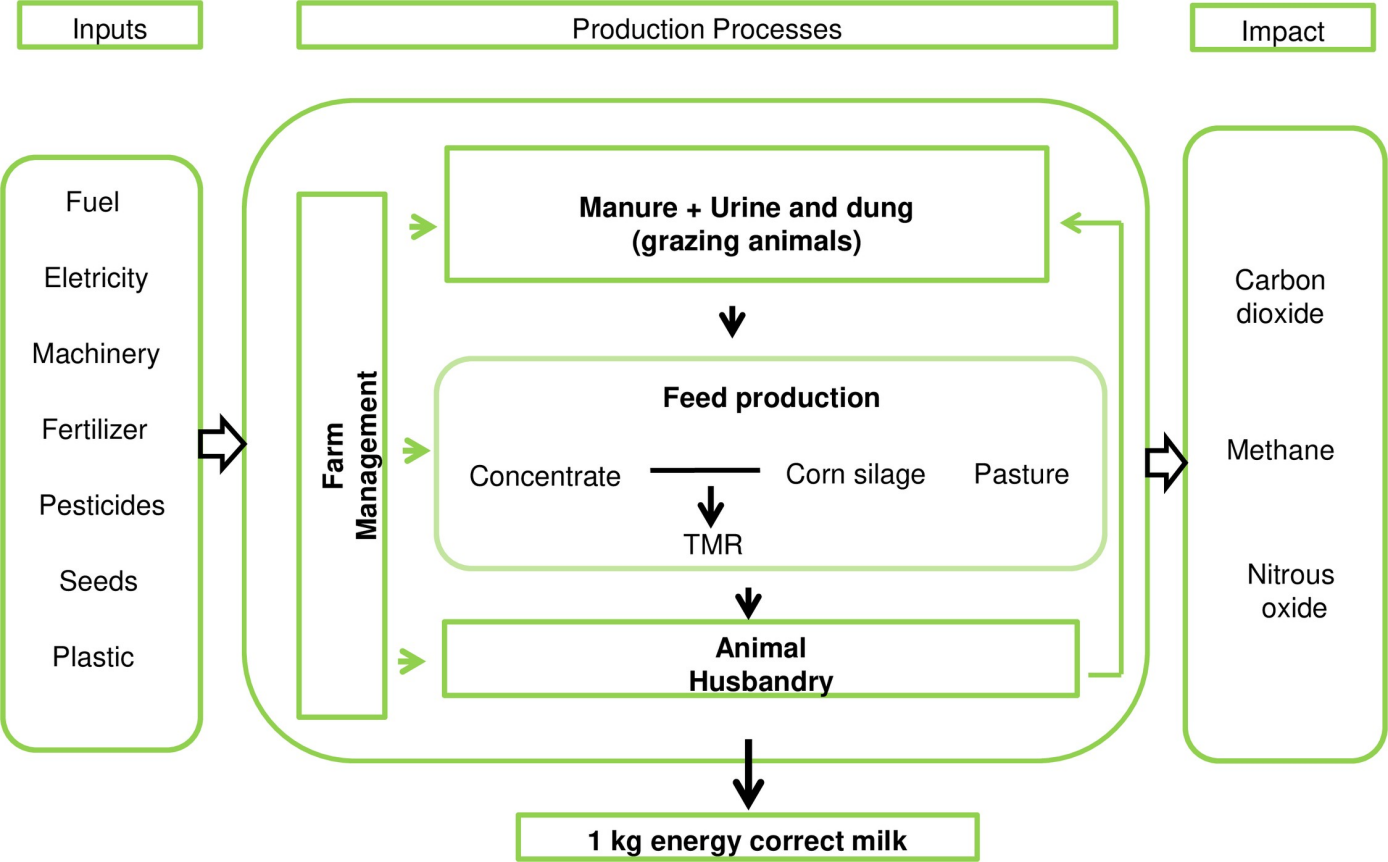
Overview of the milk production system boundary considered in the study.

The goal of the study was to assess the C footprint of annual tropical and temperate pastures in lactating dairy cow diets. The production system was divided into four main processes: (i) animal husbandry, (ii) manure management and urine and dung deposited by grazing animals, (iii) production of feed ingredients and (iv) farm management (Fig 1). The study boundary included all processes up to the animal farm gate (cradle to gate), including secondary sources such as GHG emissions during the production of fuel, electricity, machinery, manufacturing of fertilizer, pesticides, seeds and plastic used in silage production. Fuel combustion and machinery (manufacture and repairs) for manure handling and electricity for milking and confinement were accounted as emissions from farm management. Emissions post milk production were assumed to be similar for all scenarios, therefore, activities including milk processing, distribution, retail or consumption were outside of the system boundary.

### Functional unit

The functional unit was one kilogram of energy-corrected milk (ECM) at the farm gate. All processes in the system were calculated based on one kilogram ECM. The ECM was calculated by multiplying milk production by the ratio of the energy content of the milk to the energy content of standard milk with 4% fat and 3.3% true protein according to NRC [20] as follows:

ECM = Milk production × (0.0929 × fat% + 0.0588× true protein% + 0.192) / (0.0929 × (4%) + 0.0588 × (3.3%) + 0.192), where fat% and protein% are fat and protein percentages in milk, respectively. The average milk production and composition were recorded from the University of Santa Catarina State (Brazil) herd, considering 165 lactations between 2009 and 2018. The herd is predominantly Holstein × Jersey cows, with key characteristics described in Table 1.

**Table 1:** Descriptive characteristics of the herd.

Item	Unit	Average
Milking cows	#	165
Milk production	kg year^-1^	7,015
Milk fat	%	4.0
Milk protein	%	3.3
Length of lactation	days	305
Body weight	kg	553
Lactations per cow	#	4
Replacement rate	%	25
Cull rate	%	25
First artificial insemination	months	16
Weaned	days	60
Mortality	%	3.0

### Data sources and livestock system description

The individual feed requirements, as well as the milk production responses based on feed strategies were based on data recorded from the herd described above and two experiments performed using lactating cows from the same herd. Due to the variation on herbage production throughout the year, feed requirements were estimated taking into consideration that livestock systems have a calving period in April, which represents the beginning of fall season in the southern Hemisphere. The experiments have shown a 10% reduction in ECM production in dairy cows that received both 75 and 50% of *ad libitum* TMR intake with access to grazing a tropical pasture (pearl-millet, *Pennisetum glaucum* ‘Campeiro’) compared to cows receiving *ad libitum* TMR intake. Cows grazing on a temperate pasture (ryegrass, *Lolium multiflorum* ‘Maximus’) did not need changes to ECM production compared to the *ad libitum* TMR intake group.

Using experimental data, three scenarios were evaluated during the lactation period: *ad libitum* TMR intake, and 75, and 50% of *ad libitum* TMR intake with access to grazing either an annual tropical or temperate pasture as a function of month ([26], Civiero et al., *in press*). From April to October (210 days) cows accessed an annual temperate pasture (ryegrass), and from November to beginning of February (95 days) cows grazed an annual tropical pasture (pearl-millet). The average annual reduction in ECM production in dairy cows with access to pastures is 3%. This value was assumed during an entire lactation period.

### Impact assessment

The CO_2_e emissions were calculated by multiplying the emissions of CO_2_, CH_4_ and N_2_O by their 100-year global warming potential (GWP_100_), based on IPCC assessment report 5 (AR5; [27]). The values of GWP_100_ are 1, 28 and 265 for CO_2_, CH_4_ and N_2_O, respectively.

### Feed production

#### Diets composition

The DM intake of each ingredient throughout the entire life of animals during lactation periods was calculated for each scenario: cows receiving only TMR, cows receiving 75% of TMR with annual pastures and cows receiving 50% of TMR with annual pastures (Table 2). In each of other phases of life (calf, heifer, dry cow), animals received the same diet, including a perennial tropical pasture (kikuyu grass, *Pennisetum clandestinum*). The DM intake of calves, heifers and dry cows was calculated assuming 2.8, 2.5 and 1.9% body weight, respectively [20]. In each case, the actual DM intake of concentrate and corn silage was recorded, and pasture DM intake was estimated by the difference between daily expected DM intake and actual DM intake of concentrate and corn silage. For lactating heifers and cows, TMR was formulated to meet the net energy for lactation (NE_L_) and metabolizable protein (MP) requirements of experimental animals, according to [28]. The INRA system was used because it is possible to estimate pasture DM intake taking into account the TMR intake, pasture management and the time of access to pasture using the GrazeIn model [29], which was integrated in the software INRAtion 4.07 (https://www.inration.educagri.fr/fr/forum.php). The nutrient intake was calculated as a product of TMR and pasture intake and the nutrient contents of TMR and pasture, respectively, which were determined in feed samples collected throughout the experiments.

**Table 2:** Dairy cows’ diets in different scenarios^a^.

	Calf		Pregnant/dry		Lactation			Weighted average		
	0–12 mo	12-AI mo	Heifer	Cow	TMR	TMR75	TMR50	TMR	TMR75	TMR50
Days	360	120	270	180	1220	1220	1220			
DM intake, kg d^-1^	3.35	6.90	10.4	11.0	18.7	17.2	17.0	13.8	12.9	12.8
Ingredients, g (kg DM)^-1^										
Ground corn	309	145	96.3	-	257	195	142	218	183	153
Soybean meal	138	22	26.7	-	143	105	76.1	109	88.0	71.0
Corn silage	149	290	85.6	-	601	451	326	393	308	237
Ann temperate pasture	184	326	257	-	-	185	337	81.3	186	273
Ann tropical pasture	-	-	107	-	-	63	119	13.4	49.1	81.0
Perenn tropical pasture	219	217	428	1000	-	-	-	186	186	186
Chemical composition, g (kg DM)^-1^										
Organic matter	935	924	913	916	958	939	924	943	932	924
Crude protein	216	183	213	200	150	170	198	175	186	202
Neutral detergent fibre	299	479	518	625	382	418	449	411	431	449
Acid detergent fibre	127	203	234	306	152	171	187	174	185	194
Ether extract	46.5	30.4	28.6	25.0	31.8	31.1	30.4	33.2	32.8	32.4
Nutritive value										
OM digestibility, %	82.1	77.9	77.1	71.9	72.4	75.0	77.2	74.8	76.3	77.6
NE_L_, Mcal (kg DM)^-1^	1.96	1.69	1.63	1.44	1.81	1.78	1.74	1.8	1.8	1.7
MP, g (kg DM)^-1^	111	93.6	97.6	90.0	95.0	102	102	97.5	102	101

^a^AI, artificial insemination; TMR, cows receiving exclusively total mixed ration; TMR75, cows receiving 75% of total mixed ration with pasture; TMR50, cows receiving 50% of total mixed ration with pasture; NE_L_, net energy for lactation; MP, metabolizable protein.

#### GHG emissions from crop and pasture production

GHG emission factors used for off- and on-farm feed production were based on literature values, and are presented in Table 3. The emission factor used for corn grain is the average of emission factors observed in different levels of synthetic N fertilization [30]. The emission factor used for soybean is based on Brazilian soybean production [31]. The emissions used for corn silage, including feed processing (cutting, crushing and mixing), and annual or perennial grass productions were 3300 and 1500 kg CO_2_e ha^-1^, respectively [32]. The DM production (kg ha^-1^) of corn silage and pastures were based on regional and locally recorded data [33–36], assuming that animals are able to consume 70% of pastures during grazing.

**Table 3:** GHG emission factors for Off- and On-farm feed production.

Feed	DM yield (kg ha^-1^)	Emission factor	Unit^a^	References
Off-farm				
Corn grain	7,500	0.316	kg CO_2_e (kg grain)^-1^	[30]
Soybean	2,200	0.186	kg CO_2_e (kg grain)^-1^	[31]
On-farm				
Corn silage^b^	16,000	0.206	kg CO_2_e (kg DM)^-1^	[32,33]
Annual ryegrass^c^	9,500	0.226	kg CO_2_e (kg DM)^-1^	[32,34]
Pearl millet^d^	11,000	0.195	kg CO_2_e (kg DM)^-1^	[32,35]
Kikuyu grass^e^	9,500	0.226	kg CO_2_e (kg DM)^-1^	[32,36]

^a^CO_2_e, carbon dioxide equivalent.

^b^Emission factor estimated as [kg CO_2_e ha^-1^: kg DM ha^-1^].

^c,d,e^Emission factors estimated as [kg CO_2_e ha^-1^: kg DM ha^-1^ × 0.7], assuming that animals are able to consume 70% of pasture during grazing.

Emissions from on-farm feed production (corn silage and pasture) were estimated using primary and secondary sources based on the actual amount of each input (Table 4). Primary sources were direct and indirect N_2_O-N emissions from organic and synthetic fertilizers and crop/pasture residues, CO_2_-C emissions from lime and urea applications, as well as fuel combustion. The direct N_2_O-N emission factor (kg (kg N input)^-1^) is based on a local study performed previously [37]. For indirect N_2_O-N emissions (kg N_2_O-N (kg NH_3_-N + NO_x_)^-1^), as well as CO_2_-C emissions from lime + urea, default values proposed by IPCC [38] were used. For perennial pastures, a C sequestration of 0.57 t ha^-1^ was used based on a 9-year study conducted in southern Brazil [39]. Due to the use of conventional tillage, no C sequestration was considered for annual pastures. The amount of fuel required was 8.9 (no-tillage) and 14.3 L ha^-1^ (disking) for annual tropical and temperate pastures, respectively [40]. The CO_2_ from fuel combustion was 2.7 kg CO_2_ L^-1^ [41]. Secondary sources of emissions during the production of fuel, machinery, fertilizer, pesticides, seeds and plastic for ensilage were estimated using emission factors described by Rotz et al. [42].

**Table 4:** GHG emissions from On-farm feed production.

Item	Corn silage	Annual temperate pasture	Annual tropical pasture	Perennial tropical pasture
DM yield, kg ha^-1^	16000	9500	11000	9500
Direct N_2_O emissions to air				
N organic fertilizer, kg ha^-1^^a^	150	180	225	225
N synthetic fertilizer	-	20	25	25
N from residual DM, kg ha^-1^^b^	70	112	129	112
Emission fator, kg N_2_O-N (kg N)^-1^^c^	0.002	0.002	0.002	0.002
kg N_2_O ha^-1^ from direct emissions	0.69	0.98	1.19	1.14
Indirect N_2_O emissions to air				
kg NH_3_-N+NO_x_-N (kg organic N)^-1^^b^	0.2	0.2	0.2	0.2
kg NH_3_-N+NO_x_-N (kg synthetic N)^-1^^b^	0.1	0.1	0.1	0.1
kg N_2_O-N (kg NH_3_-N+NO_x_-N)^-1^^b^	0.01	0.01	0.01	0.01
kg N_2_O ha^-1^ from NH_3_+NO_x_ volatilized	0.47	0.60	0.75	0.75
Indirect N_2_O emissions to soil				
kg N losses by leaching (kg N)^-1^^b^	0.3	0.3	0.3	0.3
kg N_2_O-N (kg N leaching)^-1^	0.0075	0.0075	0.0075	0.0075
kg N_2_O ha^-1^ from N losses by leaching	0.78	1.10	1.34	1.28
kg N_2_O ha^-1^ (direct + indirect emissions)	1.94	2.68	3.28	3.16
kg CO_2_e ha^-1^ from N_2_0 emissions^d^	514	710	869	838
kg CO_2_ ha^-1^ from lime+urea^b^	515	721	882	852
kg CO_2_ ha^-1^ from diesel combustion^e^	802	38	23	12
kg CO_2_e from secondary sources^f^	516	205	225	284
Total CO_2_e emitted, kg ha^-1^	1833	964	1130	1148
Emission factor, kg CO_2_e (kg DM)^-1^^g^	0.115	0.145	0.147	0.173
Carbon sequestered, kg ha^-1^^h^	-	-	-	570
Sequestered CO_2_-C, kg ha^-1^	-	-	-	1393
kg CO_2_e ha^-1^ (emitted—sequestered)	1833	964	1130	-245
Emission factor, kg CO_2_e (kg DM)^-1^^i^	0.115	0.145	0.147	-0.037

^a^100% of N requirements for corn silage and 90% for pastures was supplied by stocked manure.

^b^From IPCC [38].

^c^From a local study [37].

^d^From Assessment report 5 (AR5; [27]).

^e^From [40,41]

^f^Emissions during the production of fuel, machinery, fertilizer, pesticides, seeds and plastic for ensilage. Estimated as described by Rotz et al. [42].

^g^Without accounting sequestered CO_2_-C due to no-tillage for perennial pasture.

^h^From [39].

^i^Accounting sequestered CO_2_-C due to no-tillage for perennial pasture.

### Animal husbandry

The CH_4_ emissions from enteric fermentation intensity (g (kg ECM)^-1^) was a function of estimated CH_4_ yield (g (kg DM intake)^-1^), actual DM intake and ECM. The enteric CH_4_ yield was estimated as a function of neutral detergent fiber (NDF) concentration on total DM intake, as proposed by Niu et al. [43], where: CH_4_ yield (g (kg DM intake)^-1^) = 13.8 + 0.185 × NDF (% DM intake).

### Manure from confined cows and urine and dung from grazing animals

The CH_4_ emission from manure (kg (kg ECM)^-1^) was a function of daily CH_4_ emission from manure (kg cow^-1^) and daily ECM (kg cow^-1^). The daily CH_4_ emission from manure was estimated according to IPCC [38], which considered daily volatile solid (VS) excreted (kg DM cow^-1^) in manure. The daily VS was estimated as proposed by Eugène et al. [44] as: VS = NDOMI + (UE × GE) × (OM/18.45), where: VS = volatile solid excretion on an organic matter (OM) basis (kg day^-1^), NDOMI = non-digestible OM intake (kg day^-1^): (1- OM digestibility) × OM intake, UE = urinary energy excretion as a fraction of GE (0.04), GE = gross energy intake (MJ day^-1^), OM = organic matter (g), 18.45 = conversion factor for dietary GE per kg of DM (MJ kg^-1^).

The OM digestibility was estimated as a function of chemical composition, using equations published by INRA [21], which takes into account the effects of digestive interactions due to feeding level, the proportion of concentrate and rumen protein balance on OM digestibility. For scenarios where cows had access to grazing, the amount of calculated VS were corrected as a function of the time at pasture. The biodegradability of manure factor (0.13 for dairy cows in Latin America) and methane conversion factor (MCF) values were taken from IPCC [38]. The MCF values for pit storage below animal confinements (> 1 month) were used for the calculation, taking into account the annual average temperature (16.6ºC) or the average temperatures during the growth period of temperate (14.4ºC) or tropical (21ºC) annual pastures, which were 31%, 26% and 46%, respectively.

The N_2_O-N emissions from urine and feces were estimated considering the proportion of N excreted as manure and storage or as urine and dung deposited by grazing animals. These proportions were calculated based on the proportion of daily time that animals stayed on pasture (7 h/24 h = 0.29) or confinement (1−0.29 = 0.71). For lactating heifers and cows, the total amount of N excreted was calculated by the difference between N intake and milk N excretion. For heifers and non-lactating cows, urinary and fecal N excretion were estimated as proposed by Reed et al. [45] (Table 3: equations 10 and 12, respectively). The N_2_O emissions from stored manure as well as urine and dung during grazing were calculated based on the conversion of N_2_O-N emissions to N_2_O emissions, where N_2_O emissions = N_2_O-N emissions × 44/28. The emission factors were 0.002 kg N_2_O-N (kg N)^-1^ stored in a pit below animal confinements, and 0.02 kg N_2_O-N (kg of urine and dung)^-1^ deposited on pasture [38]. The indirect N_2_O emissions from storage manure and urine and dung deposits on pasture were also estimated using the IPCC [38] emission factors.

### Farm management

Emissions due to farm management included those from fuel and machinery for manure handling and electricity for milking and confinement (Table 5). Emissions due to feed processing such as cutting, crushing, mixing and distributing, as well as secondary sources of emissions during the production of fuel, machinery, fertilizer, pesticides, seeds and plastic for ensilage were included in ‘Emissions from crop and pasture production’ section.

**Table 5:** Factors for major resource inputs in farm management.

Item	Factor	Unit^a^	References
Production and transport of diesel	0.374	kg CO_2_e L^-1^	[41]
Emissions from diesel fuel combustion	2.637	kg CO_2_e L^-1^	[41]
Production of electricity^b^	0.73	kg CO_2_e kWh^-1^	[41]
Production of electricity (alternative)^c^	0.205	kg CO_2_e kWh^-1^	[46]
Production of machinery	3.54	kg CO_2_e (kg mm)^-1^	[42]
Manure handling			
Fuel for manure handling	0.600	L diesel tonne^-1^	[42]
Machinery for manure handling	0.17	kg mm kg^-1^	[42]
Milking and confinement			
Electricity for milking	0.06	kWh (kg milk)^-1^	[47]
Electricity for lighting^d^	75	kWh cow^-1^	[47]

^a^mm, machinery mass

^b^Based on United States data.

^c^Based on the Brazilian electricity matrix.

^d^Naturally ventilated barns.

The amount of fuel use for manure handling were estimated taking into consideration the amount of manure produced per cow and the amounts of fuel required for manure handling (L diesel t^-1^) [42]. The amount of manure was estimated from OM excretions (kg cow^-1^), assuming that the manure has 8% ash on DM basis and 60% DM content. The OM excretions were calculated by NDOMI × days in confinement × proportion of daily time that animals stayed on confinement.

The emissions from fuel were estimated considering the primary (emissions from fuel burned) and secondary (emissions for producing and transporting fuel) emissions. The primary emissions were calculated by the amount of fuel required for manure handling (L) × (kg CO_2_e L^-1^) [41]. The secondary emissions from fuel were calculated by the amount of fuel required for manure handling × emissions for production and transport of fuel (kg CO_2_e L^-1^) [41]. Emissions from manufacture and repair of machinery for manure handling were estimated by manure produced per cow (t) × (kg machinery mass (kg manure)^-1^ × 10^−3^) [42] × kg CO_2_e (kg machinery mass)^-1^ [42].

Emissions from electricity for milking and confinement were estimated using two emission factors (kg CO_2_ kWh^-1^). The first one is based on United States electricity matrix [41], and was used as a reference of an electricity matrix with less hydroelectric power than the region under study. The second is based on the Brazilian electricity matrix [46]. The electricity required for milking activities is 0.06 kWh (kg milk produced)^-1^ [47]. The annual electricity use for lighting was 75 kWh cow^-1^, which is the value considered for lactating cows in naturally ventilated barns [47].

### Co-product allocation

The C footprint for milk produced in the system was calculated using a biophysical allocation approach, as recommended by the International Dairy Federation [49], and described by Thoma et al. [48]. Briefly, AR_milk_ = 1–6.04 × BMR, where_:_ AR_milk_ is the allocation ratio for milk and BMR is cow BW at the time of slaughter (kg) + calf BW sold (kg) divided by the total ECM produced during cow`s entire life (kg). The AR_milk_ were 0.854 and 0.849 for TMR and TMR with both pasture scenarios, respectively. The AR_milk_ was applied to the whole emissions, except for the electricity consumed for milking (milking parlor) and refrigerant loss, which was directly assigned to milk production.

### Sensitivity analysis

A sensitivity index was calculated as described by Rotz et al. [42]. The sensitivity index was defined for each emission source as the percentage change in the C footprint for a 10% change in the given emission source divided by 10%. Thus, a value near 0 indicates a low sensitivity, whereas an index near or greater than 1 indicates a high sensitivity because a change in this value causes a similar change in the footprint.

## Results and discussion

The study has assessed the impact of tropical and temperate pastures in dairy cows fed TMR on the C footprint of dairy production in subtropics. Different factors were taken in to consideration to estimate emissions from manure (or urine and dung) of grazing animals, feed production and electricity use.

### Greenhouse gas emissions

**Fig 2:**
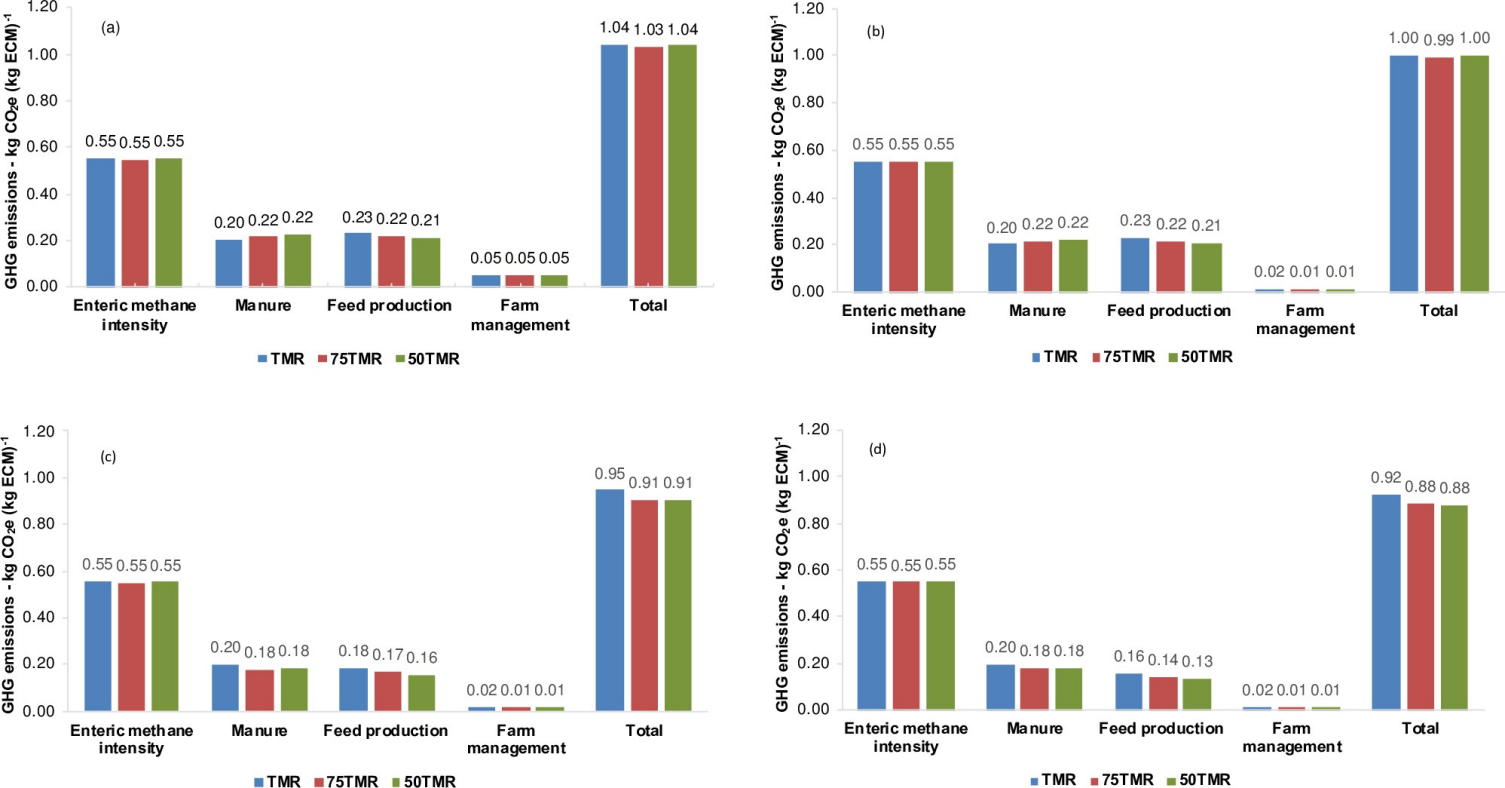
Overall greenhouse gas emissions in dairy cattle systems under various scenarios. TMR = *ad libitum* TMR intake, 75TMR = 75% of *ad libitum* TMR intake with access to pasture, 50TMR = 50% of *ad libitum* TMR intake with access to pasture. (a) N_2_O emission factors for urine and dung from IPCC [38], feed production emission factors from Table 3 without accounting for sequestered CO_2_-C from perennial pasture, production of electricity = 0.73 kg CO_2_e kWh^-1^ [41]. (b) N_2_O emission factors for urine and dung from IPCC [38], feed production emission factors from Table 3 without accounting for sequestered CO_2_-C from perennial pasture, production of electricity = 0.205 kg CO_2_e kWh^-1^ [46]; (c) N_2_O emission factors for urine and dung from local data [37], feed production EF from Table 4 without accounting for sequestered CO_2_-C from perennial pasture, production of electricity = 0.205 kg CO_2_e kWh^-1^ [46]. (d) N_2_O emission factors for urine and dung from local data [37], feed production emission factors from Table 4 accounting for sequestered CO_2_-C from perennial pasture, production of electricity = 0.205 kg CO_2_e kWh^-1^ [46].

Depending on emission factors used for calculating emissions from urine and dung (IPCC or local data) and feed production (Tables 3 or 4), the C footprint was similar (Fig 2A and 2B) or decreased by 0.04 kg CO_2_e (kg ECM)^-1^ (Fig 2C and 2D) in scenarios that included pastures compared to *ad libitum* TMR intake. Due to differences in emission factors, the overall GHG emission values ranged from 0.92 to 1.04 kg CO_2_e (kg ECM)^-1^ for dairy cows receiving TMR exclusively, and from 0.88 to 1.04 kg CO_2_e (kg ECM)^-1^ for cows with access to pasture. Using IPCC emission factors [38], manure emissions increased as TMR intake went down (Fig 2A and 2B). However, using local emission factors for estimating N_2_O-N emissions [37], manure emissions decreased as TMR intake went down (Fig 2C and 2D). Regardless of emission factors used (Tables 3 or 4), emissions from feed production decreased to a small extent as the proportion of TMR intake decreased. Emissions from farm management did not contribute more than 5% of overall GHG emissions.

Considering IPCC emission factors for N_2_O emissions from urine and dung [38] and those from Table 3, the C footprint ranged from 0.99 to 1.04 kg CO_2_e (kg ECM)^-1^, and was close to those reported under confined based systems in California [49], Canada [50], China [8], Ireland [9], different scenarios in Australia [51,52] and Uruguay [11], which ranged from 0.98 to 1.16 kg CO_2_e (kg ECM)^-1^. When local emission factors for N_2_O emissions from urine and dung [37] and those from Table 4 were taking into account, the C footprint for scenarios including pasture, without accounting for sequestered CO_2_-C from perennial pasture—0.91 kg CO_2_e (kg ECM)^-1^—was lower than the range of values described above. However, these values were still greater than high-performance confinement systems in UK and USA [53] or grass based dairy systems in Ireland [9,53] and New Zealand [8,54], which ranged from 0.52 to 0.89 kg CO_2_e (kg ECM)^-1^. Regardless of which emission factor was used, we found a lower C footprint in all conditions compared to scenarios with lower milk production per cow or in poor conditions of manure management, which ranged from 1.4 to 2.3 kg CO_2_e (kg ECM)^-1^ [8,55]. Thus, even though differences between studies may be partially explained by various assumptions (e.g., emission factors, co-product allocation, methane emissions estimation, sequestered CO_2_-C, etc.), herd productivity and manure management were systematically associated with the C footprint of the dairy systems.

The similarity of C footprint between different scenarios using IPCC [38] for estimating emissions from manure and for emissions from feed production (Table 3) was a consequence of the trade-off between greater manure emissions and lower emissions to produce feed, as the proportion of pasture in diets increased. Additionally, the small negative effect of pasture on ECM production also contributed to the trade-off. The impact of milk production on the C footprint was reported in a meta-analysis comprising 30 studies from 15 different countries [22]. As observed in this study (Fig 2A and 2B) the authors reported no significant difference between the C footprint of pasture-based vs. confinement systems. However, they observed that an increase of 1000 kg cow^-1^ (5000 to 6000 kg ECM) reduced the C footprint by 0.12 kg CO_2_e (kg ECM)^-1^, which may explain an apparent discrepancy between our study and an LCA performed in south Brazilian conditions [56]. Their study compared a confinement and a grazing-based dairy system with annual average milk production of 7667 and 5535 kg cow, respectively. In this study, the same herd was used in all systems, with an annual average milk production of around 7000 kg cow^-1^. Experimental data showed a reduction not greater than 3% of ECM when 50% of TMR was replaced by pasture access.

The lower C footprint in scenarios with access to pasture, when local emission factors [37] were used for N_2_O emissions from urine and dung and for feed production (Table 4), may also be partially attributed to the small negative effect of pasture on ECM production. Nevertheless, local emission factors for urine and dung had a great impact on scenarios including pastures compared to *ad libitum* TMR intake. Whereas the IPCC [38] considers an emission of 0.02 kg N_2_O-N (kg N)^-1^ for urine and dung from grazing animals, experimental evidence shows that it may be up to five times lower, averaging 0.004 kg N_2_O-N kg^-1^ [37].

### Methane emissions

**Fig 3:**
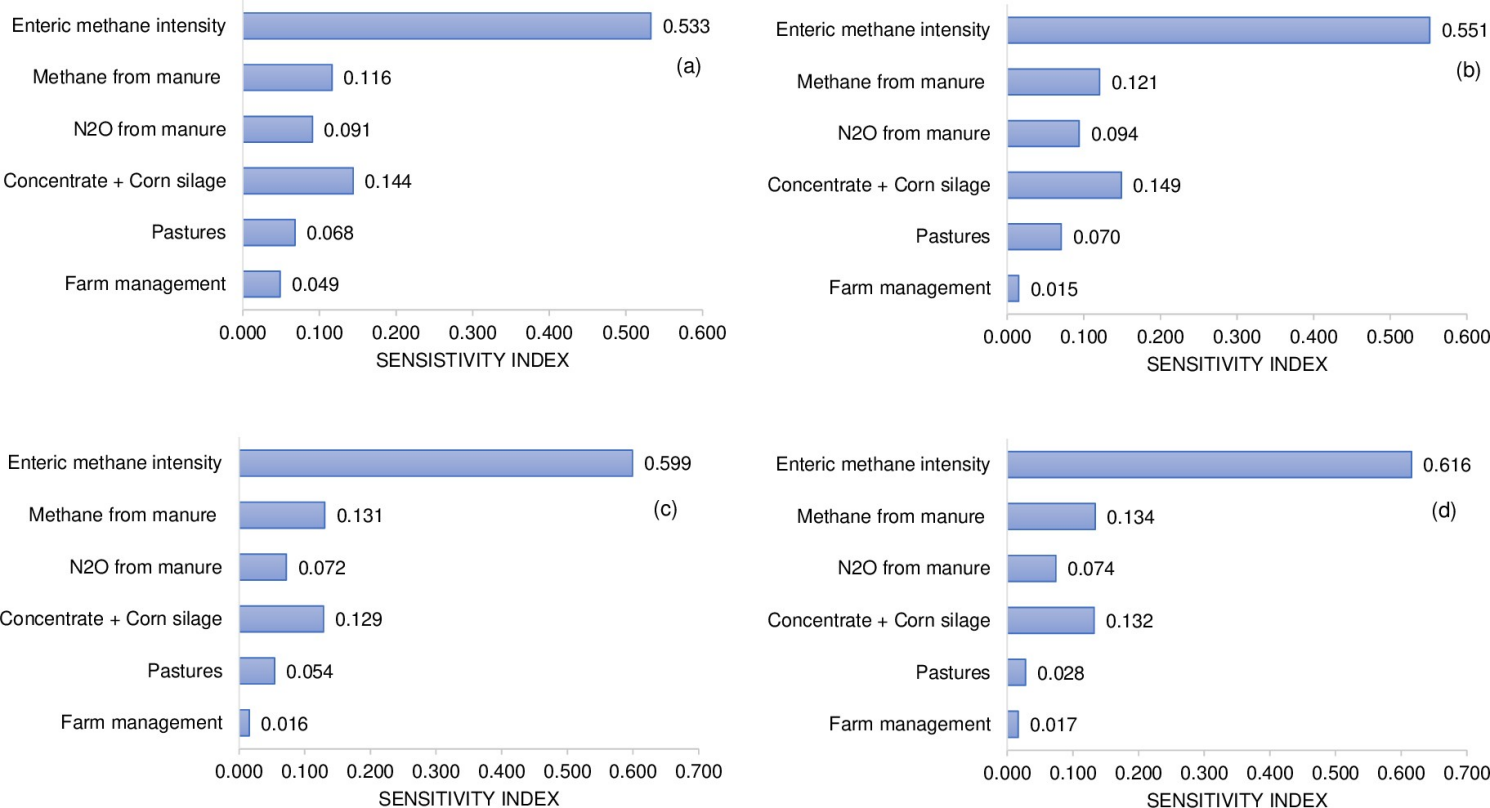
Sensitivity of the C footprint. Sensitivity index = percentage change in C footprint for a 10% change in the given emission source divided by 10% of. (a) N_2_O emission factors for urine and dung from IPCC [38], feed production emission factors from Table 3, production of electricity = 0.73 kg CO_2_e kWh^-1^ [41]. (b) N_2_O emission factors for urine and dung from IPCC [38], feed production emission factors from Table 3, production of electricity = 0.205 kg CO_2_e kWh^-1^ [46]; (c) N_2_O emission factors for urine and dung from local data [37], feed production EF from Table 4 without accounting sequestered CO_2_-C from perennial pasture, production of electricity = 0.205 kg CO_2_e kWh^-1^ [46]. (d) N_2_O emission factors for urine and dung from local data [37], feed production emission factors from Table 4 accounting sequestered CO_2_-C from perennial pasture, production of electricity = 0.205 kg CO_2_e kWh^-1^ [46].

The enteric CH_4_ intensity was similar between different scenarios (Fig 2), showing the greatest sensitivity index, with values ranging from 0.53 to 0.62, which indicate that for a 10% change in this source, the C footprint may change between 5.3 and 6.2% (Fig 3). The large effect of enteric CH_4_ emissions on the whole C footprint was expected, because the impact of enteric CH_4_ on GHG emissions of milk production in different dairy systems has been estimated to range from 44 to 60% of the total CO_2_e [50,52,57,58]. However, emissions in feed production may be the most important source of GHG when emission factors for producing concentrate feeds are greater than 0.7 kg CO_2_e kg^-1^ [59], which did not happen in this study.

The lack of difference in enteric CH_4_ emissions in different systems can be explained by the narrow range of NDF content in diets (<4% difference). This non-difference is due to the lower NDF content of annual temperate pastures (495 g (kg DM)^-1^) compared to corn silage (550 g (kg DM)^-1^). Hence, an expected, increase NDF content with decreased concentrate was partially offset by an increase in the pasture proportion relatively low in NDF. This is in agreement with studies conducted in southern Brazil, which have shown that the actual enteric CH_4_ emissions may decrease with inclusion of temperate pastures in cows receiving corn silage and soybean meal [60] or increase enteric CH_4_ emissions when dairy cows grazing a temperate pasture was supplemented with corn silage [61]. Additionally, enteric CH_4_ emissions did not differ between dairy cows receiving TMR exclusively or grazing a tropical pasture in the same scenarios as in this study [26].

### Emissions from excreta and feed production

**Fig 4:**
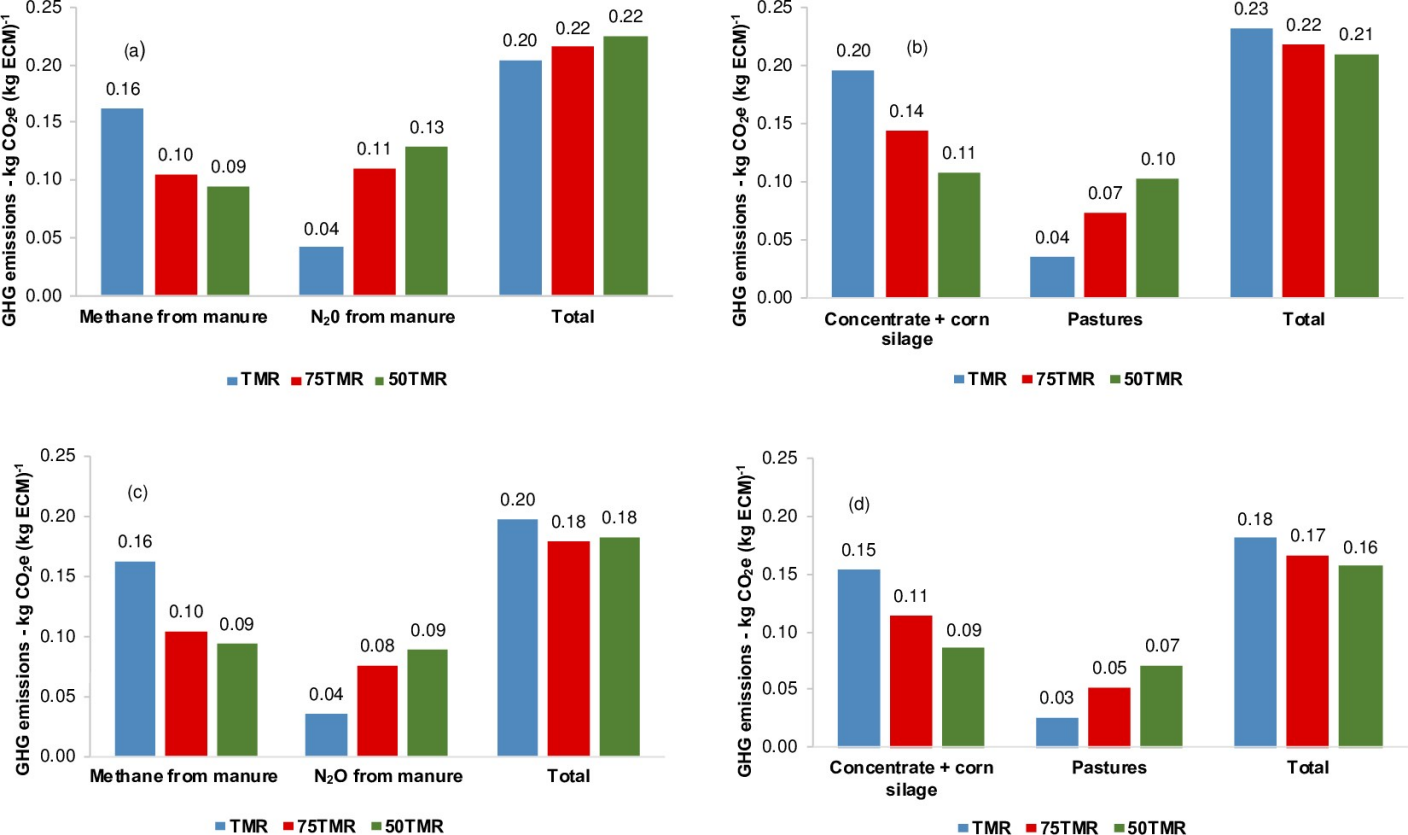
Greenhouse gas emissions (GHG) from manure and feed production in dairy cattle systems. TMR = *ad libitum* TMR intake, 75TMR = 75% of *ad libitum* TMR intake with access to pasture, 50TMR = 50% of *ad libitum* TMR intake with access to pasture. (a) N_2_O emission factors for urine and dung from IPCC [38]. (b) Feed production emission factors from Table 3. (c) N_2_O emission factors for urine and dung from local data [37]. (d) Feed production emission factors from Table 4 accounting sequestered CO_2_-C from perennial pasture.

Using IPCC emission factors for N_2_O emissions from urine and dung [38] and those from Table 3, CH_4_ emissions from manure decreased 0.07 kg CO_2_e (kg ECM)^-1^, but N_2_O emissions from manure increased 0.09 kg CO_2_e (kg ECM)^-1^, as TMR intake was restricted to 50% *ad libitum* (Fig 4A). Emissions for pastures increased by 0.06 kg CO_2_e (kg ECM)^-1^, whereas emissions for producing concentrate feeds and corn silage decreased by 0.09 kg CO_2_e (kg ECM)^-1^, as TMR intake decreased (Fig 4B). In this situation, the lack of difference in calculated C footprints of different systems was also due to the greater emissions from manure, and offset by lower emissions from feed production with inclusion of pasture in lactating dairy cow diets. The greater N_2_O-N emissions from manure with pasture was a consequence of higher N_2_O-N emissions due to greater CP content and N urine excretion, as pasture intake increased. The effect of CP content on urine N excretion has been shown by several authors in lactating dairy cows [62–64]. For instance, by decreasing CP content from 185 to 152 g (kg DM)^-1^, N intake decreased by 20% and urine N excretion by 60% [62]. In this study, the CP content for lactating dairy cows ranged from 150 g (kg DM)^-1^ on TMR system to 198 g (kg DM)^-1^ on 50% TMR with pasture. Additionally, greater urine N excretion is expected with greater use of pasture. This occurs because protein utilization in pastures is inefficient, as the protein in fresh forages is highly degradable in the rumen and may not be captured by microbes [65].

Using local emission factors for N_2_O emissions from urine and dung [37] and those from Table 4, reductions in CH_4_ emissions from stocked manure, when pastures were included on diets, did not offset by increases in N_2_O emissions from excreta (Fig 4C). In this case, total emissions from manure (Fig 4C) and feed production (Fig 4D) decreased with the inclusion of pasture. The impact of greater CP content and N urine excretion with increased pasture intake was offset by the much lower emission factors used for N_2_O emissions from urine and dung. As suggested by other authors [66,67], these results show that IPCC default value may need to be revised for the subtropical region.

Emissions for feed production decreased when pasture was included due to the greater emission factor for corn grain production compared to pastures. Emissions from concentrate and silage had at least twice the sensitivity index compared to emissions from pastures. The amount of grain required per cow in a lifetime decreased from 7,300 kg to 4,000 kg when 50% of TMR was replaced by pasture access. These results are in agreement with other studies which found lower C footprint, as concentrate use is reduced and/or pasture is included [9,68,69]. Moreover, it has been demonstrated that in intensive dairy systems, after enteric fermentation, feed production is the second main contributor to C footprint [50]. There is potential to decrease the environmental impact of dairy systems by reducing the use of concentrate ingredients with high environmental impact, particularly in confinements [9].

### Farm management

The lower impact of emissions from farm management is in agreement with other studies conducted in Europe [9, 62] and USA [42, 55], where the authors found that most emissions in dairy production systems are from enteric fermentation, feed production and emissions from excreta. As emissions from fuel for on-farm feed production were accounted into the ‘emissions from crop and pasture production’, total emissions from farm management were not greater than 5% of total C footprint.

Emissions from farm management dropped when the emission factor for electricity generation was based on the Brazilian matrix. In this case, the emission factor for electricity generation (0.205 kg CO_2_e kWh^-1^ [46]) is much lower than that in a LCA study conducted in US (0.73 kg CO_2_e kWh^-1^ [42]). This apparent discrepancy is explained because in 2016, almost 66% of the electricity generated in Brazil was from hydropower, which has an emission factor of 0.074 kg CO_2_e kWh^-1^ against 0.382 and 0.926 kg CO_2_e kWh^-1^ produced by natural gas and hard coal, respectively [46].

### Assumptions and limitations

The milk production and composition data are the average for a typical herd, which might have great animal-to-animal variability. Likewise, DM yield of crops and pastures were collected from experimental observations, and may change as a function of inter-annual variation, climatic conditions, soil type, fertilization level etc. The emission factors for direct and indirect N_2_O emissions from urine and dung were alternatively estimated using local data, but more experiments are necessary to reduce the uncertainty. The CO_2_ emitted from lime and urea application was estimated from IPCC default values, which may not represent emissions in subtropical conditions. This LCA may be improved by reducing the uncertainty of factors for estimating emissions from excreta and feed production, including the C sequestration or emissions as a function of soil management.

### Further considerations

The potential for using pasture can reduce the C footprint because milk production kept pace with animal confinement. However, if milk production is to decrease with lower TMR intake and inclusion of pasture [19], the C footprint would be expected to increase. Lorenz et al. [22] showed that an increase in milk yield from 5,000 to 6,000 kg ECM reduced the C footprint by 0.12 kg CO_2_e (kg ECM)^-1^, whereas an increase from 10,000 to 11,000 kg ECM reduced the C footprint by only 0.06 kg CO_2_e (kg ECM)^-1^. Hence, the impact of increasing milk production on decreasing C footprint is not linear, and mitigation measures, such as breeding for increased genetic yield potential and increasing concentrate ratio in the diet, are potentially harmful for animal’s health and welfare [70]. For instance, increasing concentrate ratio potentially increases the occurrence of subclinical ketosis and foot lesions, and C footprint may increase by 0.03 kg CO_2_e (kg ECM)^-1^ in subclinical ketosis [71] and by 0.02 kg CO_2_e (kg ECM)^-1^ in case of foot lesions [72].

Grazing lands may also improve biodiversity [73]. Strategies such as zero tillage may increase stocks of soil C [74]. This study did not consider C sequestration during the growth of annual pastures, because it was assumed these grasses were planted with tillage, having a balance between C sequestration and C emissions [38]. Considering the C sequestration from no-tillage perennial pasture, the amount of C sequestration will more than compensates for C emitted. These results are in agreement with other authors who have shown that a reduction or elimination of soil tillage increases annual soil C sequestration in subtropical areas by 0.5 to 1.5 t ha^-1^ [75]. If 50% of tilled areas were under perennial grasslands, 1.0 t C ha^-1^ would be sequestered, further reducing the C footprint by 0.015 and 0.025 kg CO_2_e (kg ECM)^-1^ for the scenarios using 75 and 50% TMR, respectively. Eliminating tillage, the reduction on total GHG emissions would be 0.03 and 0.05 kg CO_2_e (kg ECM)^-1^ for 75 and 50% TMR, respectively. However, this approach may be controversial because lands which have been consistently managed for decades have approached steady state C storage, so that net exchange of CO_2_ would be negligible [76].

## Conclusions

This study assessed the C footprint of dairy cattle systems with or without access to pastures. Including pastures showed potential to maintain or decrease to a small extent the C footprint, which may be attributable to the evidence of low N_2_O emissions from urine and dung in dairy systems in subtropical areas. Even though the enteric CH_4_ intensity was the largest source of CO_2_e emissions, it did not change between different scenarios due to the narrow range of NDF content in diets and maintaining the same milk production with or without access to pastures.
